# 5-Chloro-3-(4-fluoro­phenyl­sulfon­yl)-2-methyl-1-benzofuran

**DOI:** 10.1107/S1600536810032502

**Published:** 2010-08-18

**Authors:** Hong Dae Choi, Pil Ja Seo, Byeng Wha Son, Uk Lee

**Affiliations:** a Department of Chemistry, Dongeui University, San 24 Kaya-dong Busanjin-gu, Busan 614-714, Republic of Korea; b Department of Chemistry, Pukyong National University, 599-1 Daeyeon 3-dong, Nam-gu, Busan 608-737, Republic of Korea

## Abstract

In the title compound, C_15_H_10_ClFO_3_S, the 4-fluoro­phenyl ring makes a dihedral angle of 75.83 (5)° with the plane of the benzofuran fragment. In the crystal, weak inter­molecular C—H⋯O hydrogen bonds link the mol­ecules into centrosymmetric dimers, which are further linked *via* an aromatic π–π inter­action between the benzene rings of adjacent mol­ecules [centroid–centroid distance = 3.510 (2) Å].

## Related literature

For the pharmacological activity of benzofuran compounds, see: Aslam *et al.* (2006 ▶); Galal *et al.* (2009 ▶); Khan *et al.* (2005 ▶). For natural products with benzofuran rings, see: Akgul & Anil (2003 ▶); Soekamto *et al.* (2003 ▶). For the structures of related 3-(4-fluoro­phenyl­sulfon­yl)-5-halogeno-2-methyl-1-benzofuran derivatives, see: Choi *et al.* (2010*a* ▶,*b* ▶).
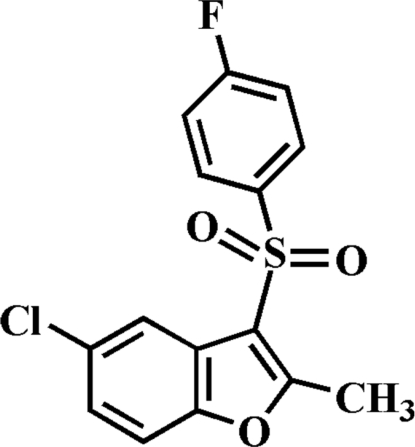


## Experimental

### 

#### Crystal data

C_15_H_10_ClFO_3_S
*M* *_r_* = 324.74
Triclinic, 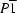

*a* = 7.341 (2) Å
*b* = 9.138 (2) Å
*c* = 11.347 (3) Å
α = 71.161 (12)°
β = 79.177 (11)°
γ = 69.108 (10)°
*V* = 670.8 (3) Å^3^
*Z* = 2
Mo *K*α radiation
μ = 0.46 mm^−1^
*T* = 173 K
0.32 × 0.32 × 0.24 mm

#### Data collection

Bruker SMART APEXII CCD diffractometer
Absorption correction: multi-scan (*SADABS*; Bruker, 2009 ▶) *T*_min_ = 0.673, *T*_max_ = 0.746
11617 measured reflections
3098 independent reflections
2847 reflections with *I* > 2σ(*I*)
*R*_int_ = 0.031

#### Refinement

*R*[*F* ^2^ > 2σ(*F* ^2^)] = 0.035
*wR*(*F* ^2^) = 0.103
*S* = 1.08
3098 reflections
191 parameters
H-atom parameters constrained
Δρ_max_ = 0.27 e Å^−3^
Δρ_min_ = −0.41 e Å^−3^

### 

Data collection: *APEX2* (Bruker, 2009 ▶); cell refinement: *SAINT* (Bruker, 2009 ▶); data reduction: *SAINT*; program(s) used to solve structure: *SHELXS97* (Sheldrick, 2008 ▶); program(s) used to refine structure: *SHELXL97* (Sheldrick, 2008 ▶); molecular graphics: *ORTEP-3* (Farrugia, 1997 ▶) and *DIAMOND* (Brandenburg, 1998 ▶); software used to prepare material for publication: *SHELXL97*.

## Supplementary Material

Crystal structure: contains datablocks global, I. DOI: 10.1107/S1600536810032502/fk2023sup1.cif

Structure factors: contains datablocks I. DOI: 10.1107/S1600536810032502/fk2023Isup2.hkl

Additional supplementary materials:  crystallographic information; 3D view; checkCIF report

## Figures and Tables

**Table 1:** Hydrogen-bond geometry (Å, °)

*D*—H⋯*A*	*D*—H	H⋯*A*	*D*⋯*A*	*D*—H⋯*A*
C11—H11⋯O3^i^	0.93	2.48	3.335 (2)	153

Symmetry code: (i) 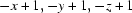.

**Fig. 1.**
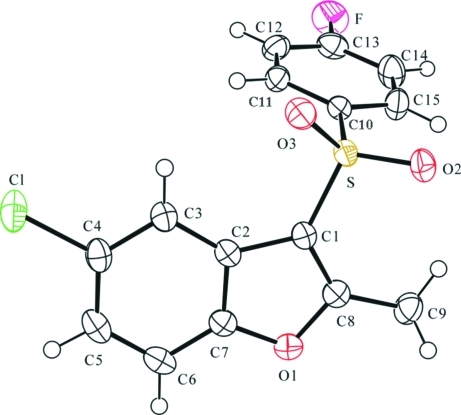
The molecular structure of the title compound with the atom numbering scheme. Displacement ellipsoids are drawn at the 50% probability level. H atoms are presented as small spheres of arbitrary radius.

**Fig. 2.**
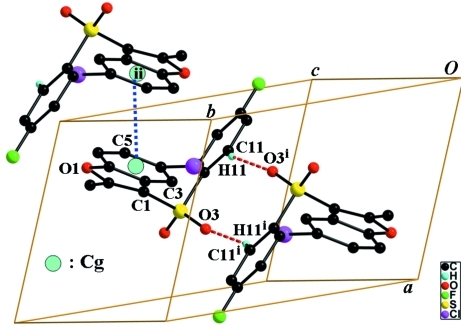
C–H···O and π–π interactions (dotted lines) in the crystal structure of the title compound. Cg denotes the ring centroid. [Symmetry codes: (i) -x+1, -y+1, -z+1; (ii) -x, -y+2, -z+1.]

## supplementary crystallographic information

### Comment

Many compounds containing a benzofuran skeleton show interesting pharmacological properties such as antifungal, antitumor, antiviral, and antimicrobial activities (Aslam *et al.*, 2006, Galal *et al.*, 2009, Khan *et al.*, 2005). These compounds widely occur in nature (Akgul & Anil, 2003; Soekamto *et al.*, 2003). As a part of our study of the substituent effect on the solid state structures of 3-(4-fluorophenylsulfonyl)-5-halo-2-methyl-1-benzofuran analogues (Choi *et al.*, 2010**a*,b*), we report the crystal structure of the title compound (Fig. 1).

The benzofuran unit is essentially planar, with a mean deviation of 0.013 (1) Å from the least-squares plane defined by the nine constituent atoms. The dihedral angle formed by the benzofuran plane and the 4-fluorophenyl ring is 75.83 (5)°. The crystal packing (Fig. 2) is stabilized by weak intermolecular C–H···O hydrogen bonds between the 4-fluorophenyl H atom and the oxygen of the O═S═O unit, with C11–H11···O3^i^ (Table 1). The packing is further stabilized by an aromatic π–π interaction between the benzene rings of neighbouring molecules, with a Cg···Cg^ii^ distance of 3.510 (2) Å (Cg is the centroid of the C2-C7 benzene ring).

### Experimental

77% 3-chloroperoxybenzoic acid (515 mg, 2.3 mmol) was added in small portions to a stirred solution of 5-chloro-3-(4-fluorophenylsulfanyl)-2-methyl-1-benzofuran (339 mg, 1.1 mmol) in dichloromethane (40 mL) at 273 K. After being stirred at room temperature for 10h, the mixture was washed with saturated sodium bicarbonate solution and the organic layer was separated, dried over magnesium sulfate, filtered and concentrated at reduced pressure. The residue was purified by column chromatography (benzene) to afford the title compound as a colourless solid [yield 78%, m.p. 452-453 K; R_f_ = 0.54 (benzene)]. Single crystals suitable for X-ray diffraction were prepared by slow evaporation of a solution of the title compound in chloroform at room temperature.

### Refinement

All H atoms were clearly located from Fourier difference maps and refined at idealized positions using a riding model, with C–H = 0.93 Å for aryl and 0.96 Å for methyl H atoms. *U*_iso_(H) = 1.2*U*_eq_(C) for aryl and 1.5*U*_eq_(C) for methyl H atoms.

### Crystal data

**Table:**

C_15_H_10_ClFO_3_S	*Z* = 2
*M**_r_* = 324.74	*F*(000) = 332
Triclinic, *P*1	*D*_x_ = 1.608 Mg m^−^^3^
Hall symbol: -P 1	Mo *K*α radiation, λ = 0.71073 Å
*a* = 7.341 (2) Å	Cell parameters from 8342 reflections
*b* = 9.138 (2) Å	θ = 2.5–27.7°
*c* = 11.347 (3) Å	µ = 0.46 mm^−^^1^
α = 71.161 (12)°	*T* = 173 K
β = 79.177 (11)°	Block, colourless
γ = 69.108 (10)°	0.32 × 0.32 × 0.24 mm
*V* = 670.8 (3) Å^3^	

### Data collection

**Table:**

Bruker SMART APEXII CCD diffractometer	3098 independent reflections
Radiation source: rotating anode	2847 reflections with *I* > 2σ(*I*)
graphite multilayer	*R*_int_ = 0.031
Detector resolution: 10.0 pixels mm^-1^	θ_max_ = 27.7°, θ_min_ = 1.9°
φ and ω scans	*h* = −9→9
Absorption correction: multi-scan (*SADABS*; Bruker, 2009)	*k* = −11→11
*T*_min_ = 0.673, *T*_max_ = 0.746	*l* = −14→14
11617 measured reflections	

### Refinement

**Table:**

Refinement on *F*^2^	Primary atom site location: structure-invariant direct methods
Least-squares matrix: full	Secondary atom site location: difference Fourier map
*R*[*F*^2^ > 2σ(*F*^2^)] = 0.035	Hydrogen site location: difference Fourier map
*wR*(*F*^2^) = 0.103	H-atom parameters constrained
*S* = 1.08	*w* = 1/[σ^2^(*F*_o_^2^) + (0.0581*P*)^2^ + 0.2203*P*] where *P* = (*F*_o_^2^ + 2*F*_c_^2^)/3
3098 reflections	(Δ/σ)_max_ < 0.001
191 parameters	Δρ_max_ = 0.27 e Å^−^^3^
0 restraints	Δρ_min_ = −0.41 e Å^−^^3^

### Special details

**Table:**

Geometry. All esds (except the esd in the dihedral angle between two l.s. planes) are estimated using the full covariance matrix. The cell esds are taken into account individually in the estimation of esds in distances, angles and torsion angles; correlations between esds in cell parameters are only used when they are defined by crystal symmetry. An approximate (isotropic) treatment of cell esds is used for estimating esds involving l.s. planes.
Refinement. Refinement of F^2^ against ALL reflections. The weighted R-factor wR and goodness of fit S are based on F^2^, conventional R-factors R are based on F, with F set to zero for negative F^2^. The threshold expression of F^2^ > 2sigma(F^2^) is used only for calculating R-factors(gt) etc. and is not relevant to the choice of reflections for refinement. R-factors based on F^2^ are statistically about twice as large as those based on F, and R- factors based on ALL data will be even larger.

### Fractional atomic coordinates and isotropic or equivalent isotropic displacement parameters (Å^2^)

**Table:**

	*x*	*y*	*z*	*U*_iso_*/*U*_eq_	
Cl	0.24730 (7)	0.91535 (6)	0.18345 (4)	0.04478 (15)	
S	0.53086 (5)	0.60366 (4)	0.70560 (3)	0.02358 (12)	
O1	0.22044 (16)	1.06562 (13)	0.64622 (10)	0.0287 (2)	
O2	0.64062 (16)	0.58741 (14)	0.80400 (10)	0.0305 (3)	
O3	0.63148 (17)	0.54999 (13)	0.59840 (10)	0.0316 (3)	
C1	0.3925 (2)	0.80676 (17)	0.65127 (13)	0.0239 (3)	
C2	0.3246 (2)	0.88838 (17)	0.52755 (13)	0.0233 (3)	
C3	0.3365 (2)	0.84457 (19)	0.41916 (14)	0.0269 (3)	
H3	0.4057	0.7394	0.4135	0.032*	
C4	0.2404 (2)	0.9649 (2)	0.32022 (14)	0.0295 (3)	
C5	0.1371 (2)	1.1238 (2)	0.32493 (15)	0.0321 (3)	
H5	0.0756	1.2006	0.2557	0.038*	
C6	0.1260 (2)	1.16749 (19)	0.43212 (16)	0.0306 (3)	
H6	0.0585	1.2731	0.4374	0.037*	
C7	0.2195 (2)	1.04713 (18)	0.53112 (14)	0.0252 (3)	
C8	0.3252 (2)	0.91825 (18)	0.71784 (14)	0.0266 (3)	
C9	0.3365 (3)	0.9110 (2)	0.84855 (15)	0.0357 (4)	
H9A	0.4135	0.8034	0.8914	0.053*	
H9B	0.2071	0.9365	0.8898	0.053*	
H9C	0.3963	0.9886	0.8492	0.053*	
C10	0.3594 (2)	0.50045 (17)	0.77249 (13)	0.0239 (3)	
C11	0.2586 (2)	0.46848 (18)	0.69636 (14)	0.0278 (3)	
H11	0.2814	0.5015	0.6099	0.033*	
C12	0.1243 (2)	0.3871 (2)	0.75089 (17)	0.0338 (4)	
H12	0.0533	0.3659	0.7022	0.041*	
C13	0.0986 (3)	0.3385 (2)	0.87841 (17)	0.0355 (4)	
C14	0.1979 (3)	0.3677 (2)	0.95508 (16)	0.0395 (4)	
H14	0.1769	0.3318	1.0415	0.047*	
C15	0.3287 (3)	0.4512 (2)	0.90095 (15)	0.0331 (3)	
H15	0.3963	0.4745	0.9505	0.040*	
F	−0.03024 (18)	0.25795 (15)	0.93220 (12)	0.0533 (3)	

### Atomic displacement parameters (Å^2^)

**Table:**

	*U*^11^	*U*^22^	*U*^33^	*U*^12^	*U*^13^	*U*^23^
Cl	0.0493 (3)	0.0579 (3)	0.0263 (2)	−0.0134 (2)	−0.01042 (18)	−0.01054 (19)
S	0.02279 (19)	0.02453 (19)	0.02076 (19)	−0.00504 (14)	−0.00356 (14)	−0.00462 (13)
O1	0.0298 (6)	0.0254 (5)	0.0308 (6)	−0.0077 (4)	−0.0021 (4)	−0.0093 (4)
O2	0.0275 (5)	0.0352 (6)	0.0283 (6)	−0.0104 (5)	−0.0088 (4)	−0.0041 (5)
O3	0.0311 (6)	0.0313 (6)	0.0261 (5)	−0.0037 (5)	0.0011 (4)	−0.0088 (4)
C1	0.0231 (7)	0.0255 (7)	0.0221 (7)	−0.0078 (5)	−0.0023 (5)	−0.0047 (5)
C2	0.0197 (6)	0.0244 (7)	0.0236 (7)	−0.0080 (5)	−0.0014 (5)	−0.0030 (5)
C3	0.0251 (7)	0.0292 (7)	0.0239 (7)	−0.0071 (6)	−0.0021 (6)	−0.0059 (6)
C4	0.0268 (7)	0.0377 (8)	0.0225 (7)	−0.0129 (6)	−0.0023 (6)	−0.0036 (6)
C5	0.0255 (7)	0.0339 (8)	0.0297 (8)	−0.0105 (6)	−0.0060 (6)	0.0038 (6)
C6	0.0254 (7)	0.0234 (7)	0.0372 (8)	−0.0072 (6)	−0.0028 (6)	−0.0011 (6)
C7	0.0221 (7)	0.0257 (7)	0.0276 (7)	−0.0100 (6)	−0.0005 (5)	−0.0053 (6)
C8	0.0240 (7)	0.0278 (7)	0.0280 (7)	−0.0095 (6)	−0.0013 (6)	−0.0070 (6)
C9	0.0400 (9)	0.0395 (9)	0.0296 (8)	−0.0107 (7)	−0.0023 (7)	−0.0150 (7)
C10	0.0254 (7)	0.0210 (6)	0.0233 (7)	−0.0046 (5)	−0.0044 (5)	−0.0053 (5)
C11	0.0281 (7)	0.0280 (7)	0.0269 (7)	−0.0040 (6)	−0.0053 (6)	−0.0108 (6)
C12	0.0331 (8)	0.0308 (8)	0.0426 (9)	−0.0080 (6)	−0.0100 (7)	−0.0156 (7)
C13	0.0322 (8)	0.0283 (8)	0.0461 (10)	−0.0143 (7)	−0.0036 (7)	−0.0050 (7)
C14	0.0460 (10)	0.0450 (10)	0.0271 (8)	−0.0225 (8)	−0.0039 (7)	−0.0002 (7)
C15	0.0391 (9)	0.0396 (9)	0.0246 (7)	−0.0186 (7)	−0.0068 (6)	−0.0052 (6)
F	0.0511 (7)	0.0503 (7)	0.0638 (8)	−0.0340 (6)	−0.0040 (6)	−0.0032 (6)

### Geometric parameters (Å, °)

**Table:**

Cl—C4	1.7388 (17)	C6—H6	0.9300
S—O3	1.4336 (11)	C8—C9	1.480 (2)
S—O2	1.4365 (11)	C9—H9A	0.9600
S—C1	1.7344 (15)	C9—H9B	0.9600
S—C10	1.7534 (16)	C9—H9C	0.9600
O1—C8	1.3619 (18)	C10—C15	1.380 (2)
O1—C7	1.3716 (19)	C10—C11	1.392 (2)
C1—C8	1.360 (2)	C11—C12	1.380 (2)
C1—C2	1.448 (2)	C11—H11	0.9300
C2—C7	1.389 (2)	C12—C13	1.367 (3)
C2—C3	1.390 (2)	C12—H12	0.9300
C3—C4	1.383 (2)	C13—F	1.341 (2)
C3—H3	0.9300	C13—C14	1.373 (3)
C4—C5	1.392 (2)	C14—C15	1.371 (2)
C5—C6	1.378 (2)	C14—H14	0.9300
C5—H5	0.9300	C15—H15	0.9300
C6—C7	1.377 (2)		
			
O3—S—O2	119.62 (7)	C1—C8—O1	110.49 (13)
O3—S—C1	107.05 (7)	C1—C8—C9	134.07 (15)
O2—S—C1	108.80 (7)	O1—C8—C9	115.40 (13)
O3—S—C10	107.97 (7)	C8—C9—H9A	109.5
O2—S—C10	107.47 (7)	C8—C9—H9B	109.5
C1—S—C10	105.01 (7)	H9A—C9—H9B	109.5
C8—O1—C7	107.07 (11)	C8—C9—H9C	109.5
C8—C1—C2	107.35 (13)	H9A—C9—H9C	109.5
C8—C1—S	126.21 (12)	H9B—C9—H9C	109.5
C2—C1—S	126.43 (11)	C15—C10—C11	121.27 (15)
C7—C2—C3	119.30 (14)	C15—C10—S	118.69 (12)
C7—C2—C1	104.34 (13)	C11—C10—S	120.04 (12)
C3—C2—C1	136.33 (14)	C12—C11—C10	119.11 (15)
C4—C3—C2	116.80 (14)	C12—C11—H11	120.4
C4—C3—H3	121.6	C10—C11—H11	120.4
C2—C3—H3	121.6	C13—C12—C11	118.22 (15)
C3—C4—C5	123.19 (15)	C13—C12—H12	120.9
C3—C4—Cl	118.60 (13)	C11—C12—H12	120.9
C5—C4—Cl	118.21 (12)	F—C13—C12	118.62 (16)
C6—C5—C4	120.04 (15)	F—C13—C14	117.88 (16)
C6—C5—H5	120.0	C12—C13—C14	123.50 (16)
C4—C5—H5	120.0	C15—C14—C13	118.32 (16)
C7—C6—C5	116.73 (14)	C15—C14—H14	120.8
C7—C6—H6	121.6	C13—C14—H14	120.8
C5—C6—H6	121.6	C14—C15—C10	119.55 (15)
O1—C7—C6	125.32 (14)	C14—C15—H15	120.2
O1—C7—C2	110.74 (13)	C10—C15—H15	120.2
C6—C7—C2	123.93 (15)		

### Hydrogen-bond geometry (Å, °)

**Table:**

*D*—H···*A*	*D*—H	H···*A*	*D*···*A*	*D*—H···*A*
C11—H11···O3^i^	0.93	2.48	3.335 (2)	153.

Symmetry codes: (i) −*x*+1, −*y*+1, −*z*+1.

## References

[bb1] Akgul, Y. Y. & Anil, H. (2003). *Phytochemistry*, **63**, 939–943. 10.1016/s0031-9422(03)00357-1 12895543

[bb2] Aslam, S. N., Stevenson, P. C., Phythian, S. J., Veitch, N. C. & Hall, D. R. (2006). *Tetrahedron*, **62**, 4214–4226.

[bb3] Brandenburg, K. (1998). *DIAMOND* Crystal Impact GbR, Bonn, Germany.

[bb4] Bruker (2009). *APEX2*, *SADABS* and *SAINT* Bruker AXS Inc., Madison, Wisconsin, USA.

[bb5] Choi, H. D., Seo, P. J., Son, B. W. & Lee, U. (2010*a*). *Acta Cryst.* E**66**, o1909. 10.1107/S1600536810025468 PMC3007400 21588241

[bb6] Choi, H. D., Seo, P. J., Son, B. W. & Lee, U. (2010*b*). *Acta Cryst.* E**66**, o2049. 10.1107/S1600536810027741 PMC3007450 21588356

[bb7] Farrugia, L. J. (1997). *J. Appl. Cryst.* **30**, 565.

[bb8] Galal, S. A., Abd El-All, A. S., Abdallah, M. M. & El-Diwani, H. I. (2009). *Bioorg. Med. Chem. Lett* **19**, 2420–2428. 10.1016/j.bmcl.2009.03.069 19345581

[bb9] Khan, M. W., Alam, M. J., Rashid, M. A. & Chowdhury, R. (2005). *Bioorg. Med. Chem* **13**, 4796–4805. 10.1016/j.bmc.2005.05.009 15964760

[bb10] Sheldrick, G. M. (2008). *Acta Cryst.* A**64**, 112–122. 10.1107/S0108767307043930 18156677

[bb11] Soekamto, N. H., Achmad, S. A., Ghisalberti, E. L., Hakim, E. H. & Syah, Y. M. (2003). *Phytochemistry*, **64**, 831–834. 10.1016/j.phytochem.2003.08.009 14559276

